# The patent landscape in the field of stem cell therapy: closing the gap between research and clinic

**DOI:** 10.12688/f1000research.123799.2

**Published:** 2023-01-03

**Authors:** Dinorah Hernández-Melchor, Esther López-Bayghen, América Padilla-Viveros

**Affiliations:** 1 Science, Technology and Society Program, . Centro de Investigación y de Estudios Avanzados del Instituto Politecnico Nacional, Mexico City, 07360, Mexico; 2 Departamento de Toxicología, Centro de Investigacion y de Estudios Avanzados del Instituto Politecnico Nacional, Mexico City, 07360, Mexico

**Keywords:** Intellectual property rights, Patent landscape, Stem-cell therapy

## Abstract

Stem cell technology is a powerful tool ready to respond to the needs of modern medicine that is experiencing rapid technological development. Given its potential in therapeutic applications, intellectual property rights (IPR) as a protection resource of knowledge are a relevant topic. Patent eligibility of stem cells has been controversial as restrictions to access the fundamental technologies open a gap between research and clinic. Therefore, we depicted the current patent landscape in the field to discuss if this approach moves forward in closing this breach by examining patent activity over the last decade from a transdisciplinary perspective. Stem cell therapeutic applications is an area of continuous growth where patent filing through the PCT is the preferred strategy. Patenting activity is concentrated in the USA, European Union, and Australia; this accumulation in a few key players leads to governance, regulation, and inequality concerns. To boost wealthiness and welfare in society - stem cell therapies' ultimate goal - while at post-pandemic recovery, critical elements in the field of IPR rise to overcome current limitations: to promote bridge builders able to connect the research and business worlds, regulatory updates, novel financing models, new vehicles (startups, spinouts, and spin-offs), and alternative figures of intellectual property.

## Introduction

Innovative scientific discoveries, such as cell therapies, are routinely used among clinicians in their medical practice. ^1^ Thus, cell therapies are powerful tools ready to respond to the needs of modern medicine. Furthermore, stem cell therapies face the challenge of improving the quality of life in rapidly aging societies. ^2^ Due to the rapid technological development cell therapies are experiencing and their high potential in therapeutic applications, intellectual property rights (IPR) as a protection resource of knowledge is a relevant topic.

Stem cells are traditionally defined as undifferentiated cells with unlimited potential to regenerate cells or tissues lost due to disease and thus restore normal function. ^2^ However, more recent studies have shown stem cells to have reparative properties, homing to injury sites and stimulating tissue repair. ^3^ Those remarkable discoveries beg the question of how they can be protected and to what extent by IPR. ^4^

The patent eligibility of stem cells – particularly those derived from human embryos and human embryonic stem cells (hESC) – has long been debated in scientific and legal communities. However, precedents established in USA courts significantly narrow the scope of patent eligibility within biotechnology. The implications of recent legal changes on stem cell patent eligibility have already been compared in the European Union (EU) against those applicable to the USA. ^5^

Current research has analyzed the challenges for patents based on human stem cells with therapeutic uses and patentability limits. ^6^^,^^7^ In addition, the comprehension of state of the art has been analyzed from the legal perspective of applicable regulations in different regions and jurisdictions (Europe, the USA, China, and Japan), considering ethical issues and relevant regulatory restrictions. ^8^

International courts have widely treated cases and controversies around patent activities in stem cell technology. An emblematic case is the controversies and legal disputes derived from a family of three patents held by the Wisconsin Alumni Research Foundation (WARF) that covered the first isolation of nonhuman primate stem cells and hESC. The court considered the claims “overly broad and restrictive inhibiting researchers' access to stem cell lines due to high licensing costs”, ^9^ falling within the “Alienation Phenomena of Biotechnology Patents,” where excessive patenting restricts researcher access to the essential technologies to go further. ^10^

While surfing the intellectual property outlook, researchers face legal uncertainties, high costs, and limitations on data sharing. ^11^ Even a diligent stem cells researcher or entity that wishes to respect IPR will face uncertainty and enormous expenses in dealing with the IPR landscape. ^11^^,^^12^ Furthermore, it is antithetic for one institution or company to hold a “universal patent” that, when licensed, provides total freedom to operate, ^13^ limiting the forthcoming of the promising industry of stem cells.

WARF's patents show how despite limitations, the patent system works in conjunction with robust, nonprofit, and primarily publicly funded scientific research institutions. ^14^ However, the field of stem cell therapies is not a bidirectional relationship between academia and private companies. Instead, it is a complex ecosystem influenced by government policies and court rulings in their respective jurisdictions, ^9^ assembled over a translational model where researchers from the benchside, health professionals from the bedside, communities of healthy populations, and patient groups work together ^15^ to boost wealthiness and welfare in society.

Controversy aside, from looking at this outlook, one question arises: is the current arrangement of the patent system a pathway for closing the gap between research and clinic? Therefore, we reviewed the patent landscape of stem cells, the primary tool of various cell therapies, over the last decade (2011-2020) to understand how the patenting activity behaved by analyzing trends in patent records and the stakeholders' contributions. On these grounds, we integrate a transdisciplinary perspective that allows researchers, decision-makers, and investors, to have a broad panorama to effectively address essential factors to enable equal access to technology, tackle the governance challenges, and provide IPR alternative strategies.

## A sight of the patent landscape in the field of stem cell therapy in the last decade

As patent data represents inventive activity, in this work, we explore innovations in stem cell therapy over the last decade by analyzing the patenting activity reported at The Lens Patents and PatentScope, the WIPO's repository. ^16^ PatentScope is an official source of information that encompasses several patent authorities with sufficient technical resources to explore state-of-the-art technology in a particular field. ^17^ The Lens Patents is a collaboration between the non-profit Cambia and Queensland University of Technology that offers one of the largest patent literature and citation indexes available. ^18^

### In the patent arena, regulation matters

We may say that WARF's patent controversy is the clearest example of how the scope of IPR and policy contexts affect stem cell technology. WARF is a nonprofit foundation that manages intellectual property generated by researchers at the University of Wisconsin at Madison. In the mid-1990s, James Thomson and coworkers developed an approach to maintain long-lasting ESC lines from two species of primates. Afterward, in 1998, the group created an analogous hESC; due to governmental prohibits for using federal funds in research with human embryos, Geron Corporation funded the research in exchange for exclusive and nonexclusive rights under patents that might result. The first application for the IPR patent family was filed by WARF in the US in 1996 and awarded in 2006. However, in 2004 the European Patent Office (EPO) refused the application on moral grounds. While in the US, the controversy around WARF's patent family was centered on technical issues alleging obviousness and lack of novelty, in Europe, WARF's efforts to protect their inventions through the European Patent Office (EPO) failed due to morality concerns as the only means to obtain hESC with the claimed method involved destroying human embryos, making the method unpatentable under the Rule 28 of the European Patent Convention. ^14^

WARF's protection strategy unleashed aggressive critics and accusations of asserting control over a primary science platform needed for health-related research. WARF was challenged by Consumer Watchdog, a California-based consumer rights organization, on the grounds of obviousness at the USPTO in 2006 before the patent was granted. However, it was dismissed. In 2007 WARF liberalized its patents' licensing by eliminating the prohibition against academic researchers from sharing WARF's hESC and extending exemption on licensing fees; by 2009, they had completed 35 licensing agreements for hESC with 27 companies. ^14^ In 2014, after the Leahy–Smith America Invents Act (AIA) was declared and one year before WARF’s patent expiration, Consumer Watchdog challenged USPTO's 2006 decision on the US Court of Appeals for the Federal Circuit appealing to the US Supreme Court's decision on Association for Molecular Pathology vs. Myriad Genetics Inc. on gene patenting. The US Court dismissed the petition again as there was no legal injury related to the patent, considering that Consumer Watchdog was never sued or threatened to be sued by WARF. ^19^

Despite the challenges, inventive activity around clinical applications of stem cells has been growing during the last three decades, reflected in the steady growth of patent filing activity in stem cells since the 1990s. ^20^ A peak and drop in the first decade of the 21st century were observed, being at a stable pace until 2010. ^11^ However, by 2010, the hype of stem cell technology had dropped: added to the ethical and sociopolitical controversy, the timespan for these technologies to reach the clinic, the slow entrance of industry into the field, and the lack of business models specifically engineered for stem cell-based therapies, and regulatory uncertainties created a hard shell for investment. ^11^^,^^13^

### Stem cell therapeutic applications are an area of continuous growth and innovation.

**Figure 1.**
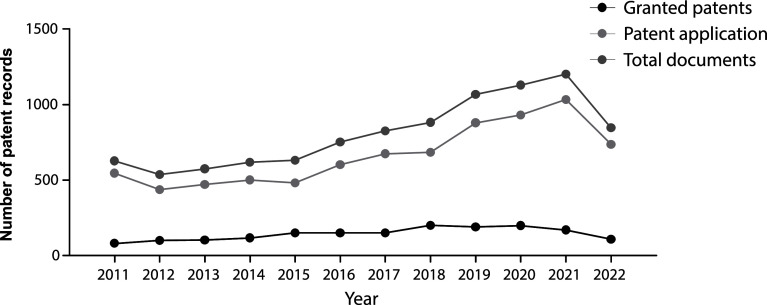
Patent documents (applications and grants) in stem cell therapies in the last decade. Own elaboration with data of The Lens Patents. ^21^

The evolution in the number of patent documents (applications and grants) from 2011 to 2020 is presented in Figure 1. From 2011 to 2015, the number of patent documents produced annually remained relatively constant. However, as of 2015, a gradual increase in annual applications and grants began, presenting a maximum peak in 2021. For 2022, the number of submitted patent applications decreased discreetly since the search results were inconclusive, considering that we did not carry out the query at the end of the year.

Against the odds, the increasing number of patent filings in the last decade mapped in our research demonstrates that the stem cell field has been an arena of continuous growth and innovation since 2015. This switch resulted logically as discourses and policies changed during the first years of the decade. For example, in 2012, the Nobel Prize in Physiology or Medicine was granted to John B. Gurdon and Shinya Yamanaka for the discovery of reprogramming: mature cells can be reprogrammed to become pluripotent, ^22^ enforcing this new avenue in stem cell therapy; also, in the US, during Obama's administration the AIA was enacted, changing the game in stem cell patent activity, making it a more attractive and competitive environment as it makes more accessible and cheaper to challenge stem cell patents as they become issued. ^19^ After that, in 2014, the EU overturned laws that banned stem cell patents, allowing patent granting if the biological materials are accurately described and have an industrial application. ^4^

### Territoriality of patent documents' filling: leading countries in the field

**Figure 2.**
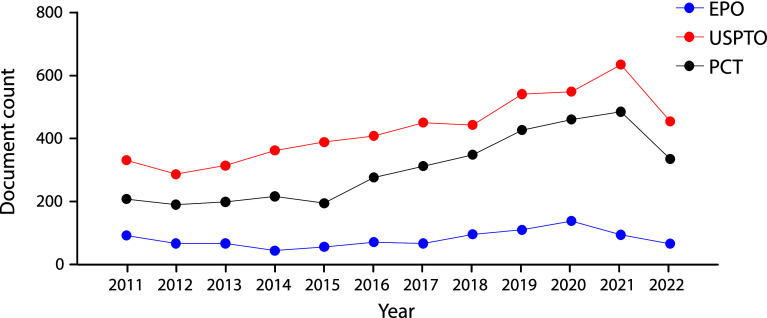
Distribution of the number of patent documents (applications and grants) in the field of stem cell therapies through different offices the Patent Cooperation Treaty (PCT) international pathway, European Patent Office (Regional), and USPTO during the last decade. Own elaboration with data of The Lens Patents. ^21^

We integrated a core collection of published patent documents constructed to encompass all stem cell applications from the USPTO, the EPO, WIPO's Patent Cooperation Treaty (PCT) filing system ( Figure 2). As in early 2000’s decade ^23^ USPTO’s filings represent the majority of global patent documents, followed by the PCT domination. The fact that patent documents are being filed through the PCT suggests the intention to protect the invention simultaneously in different countries.

**Figure 3.**
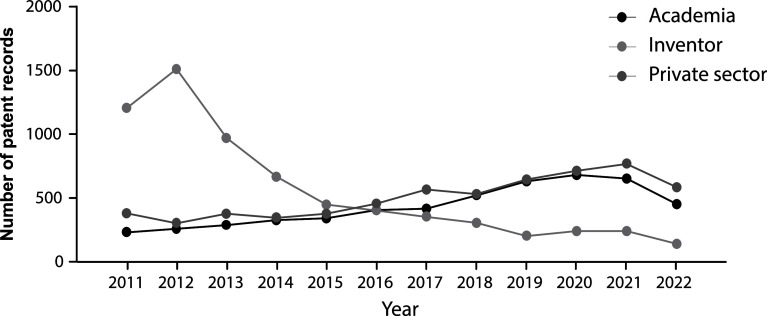
Distribution of the number of patent documents (applications and grants) in the field of stem cell therapies through different jurisdictions throughout the last decade. Own elaboration with data of The Lens Patents. ^21^

When stratified by filing national offices ( Figure 3), the United States of America remain as the most prominent target market, followed by the EPO. Hungary, United Kingdom, Israel, Canada, and China place as the leading markets in the patent arena. Other jurisdictions with less than 10 patent documents in the studied time frame include Asian (Republic of Korea, Japan), European, (Turkey, Denmark, Finland, Sweden, Ireland, Belgium, Estonia, Poland, Portugal), and Latin American countries, as well as Egypt, New Zealand, and the Russian Federation.

The United Kingdom National Stem Cell Network (UK NSC) patent watch landscape is a dataset of published patent applications and granted patents to provide a bigger picture regarding the patenting of stem cell technology. However, the UK NSC patent watches dataset is limited to published applications with WO, US, EP, and GB designations, and the granted patents on the USA, EP, and GB. Hence, to place the results of the UK patent watching a more global context and to give a fuller picture of the worldwide activity concerning stem cells, an overview of the complete global dataset would be beneficial given the recent rise in worldwide patent filings from countries such as China and India. ^24^

It is worth to mention that both USA and European Union have made changes throughout the last years to include stem cell-based and other advanced therapies within their regulatory framework, which not only contribute to understand the rise in patenting activities after 2015. The US 21st Century Cures Act (2016) considers stem cell product commercialization under the Regenerative Medicine Advanced Therapy (RMAT) designation. RMAT designation allows FDA accelerated approval based on a surrogate endpoint, such as clinical benefit. A drug is eligible for RMAT if three conditions are fulfilled (1) it is a regenerative medicine therapy (cell therapy, therapeutic tissue engineering product, human cell and tissue product, or any combination product), (2) “intended to treat, modify, reverse, or cure a serious or life-threatening disease or condition”, and (3) potential to address unmet medical needs is demonstrated by preliminary clinical evidence. ^25^ Minimally manipulated cellular products are exempt from this regulation under the Code of Federal Regulation (CFR) Title 21 1271.10(a)(1). ^26^ On the other hand, in the European Union stem cell therapeutical products are encompassed within the Advanced Therapy Medicinal Products (ATMP) classification (2007) under the Priority Medicines (PRIME) designation (2016). PRIME designation is granted by EMA to medicines that target public health unmet medical needs by offering “early and proactive support to medicine developers to optimize the generation of robust data on a medicine’s benefits and risks and enable accelerated assessment of medicines applications”. ^27^

Japan has been a substantial actor in the stem cell patent during the last decades. ^19^^,^^21^^,^^28^ Even if not listed in our research among the top 5 most active, Japan's policies have fostered stem cell innovation. Liberal Democratic Party, elected in 2012, strongly supported research in the field with over 220 million US dollars as part of a stimulus package to lift the Japanese economy from recession. ^29^ In Japan’s regulatory framework stem cell therapies are considered within three instances: the Sakigake designation, the Act on the Safety of Regenerative Medicine (RM Act), and the Pharmaceuticals and Medical Devices Act (PMD Act). ^30^^,^^31^ First, Sakigake designation (2015) is granted by Japan’s Ministry of Health, Labour and Welfare (MHLW) through the Pharmaceuticals and Medical Devices Agency (PMDA) for innovative products with intended use for a serious disease with significant impact on life and prominent effectiveness, planned to be submitted of premarket application firstly in Japan, with consideration of its development in Japan from an early stage. ^30^ Second, the RM Act encompass the use of processed cells for clinical research and medical practice guiding its administration to patients, comparable to US CFR Title 21, and considers a licensing scheme to stablish cell processing facilities outside health institutions. ^31^ Last, the PMD Act provides a specific regulatory framework to commercialize regenerative medicine products by granting time-limited marketing approval demonstrated probable benefit and proven safety. ^31^

Altogether, stem cell regulatory frameworks in US, EU, and Japan incentives innovation by favoring the commercialization of cell-based alternative therapeutical approaches trough the development of mechanisms to accelerate the bench to bed translational process. This strategy is intended to allow patients to reach these therapies earlier and significantly improve their quality of life while protecting them from harmful unapproved or unproven interventions. ^32^^,^^33^ Nevertheless, the rapidly changing in regulatory pathways raise new questions in scientific, ethical, and medical fields that are still under debate. ^34^ Through time, these storylines change the relevance of policy designs, reinforcing the notion that patent law, national policies (Economic, Educational, and Science-Technology-and-Innovation) affect how novel technologies are fostered to attain wellbeing in society.

### Universities and private companies are the leading players in the field

Table 1 presents the institutions and private corporations with more patent productivity on stem cell therapies - with more than 80 contributions each. In this top ten, seven applicants belong to academia, and four can be classified as private corporations. Four institutions are highly prestigious USA universities within academia, and two are research centers (Memorial Sloan-Kettering Cancer Center, Fred Hutchinson Cancer Center). In addition, three organizations are large pharmaceutical companies (Fred Hutchinson Cancer Center). In addition, three organizations are large pharmaceutical companies (Immunomedics Inc., Regeneron Pharmaceuticals, and Novartis Ag) and a research-focused hospital (The General Hospital Corporation DBA Massachusetts General Hospital).

**Table 1.** The ten most productive and influential organizations in patents on stem cell therapies. Own elaboration with data of The Lens Patents. ^21^

	Applicant	Number of patents documents	Type of institution
1	University of California	330	University
2	Immunomedics Inc (Undergone merge and adquisition by Gilead Sciences in 2020)	250	Private corporation
3	University of Texas	154	University
4	Memorial Sloan Kettering Cancer Center	136	Research Center
5	Leland Stanford Junior University	114	University
6	The General Hospital Corporation DBA Massachusetts General Hospital	109	Private corporation
7	Regeneron Pharmaceuticals	97	Private corporation
8	Hutchinson Fred Cancer Research Center	91	Research center
9	Novartis Ag	86	Private corporation
10	University of Pennsylvania	84	University

Interestingly, the largest biomedical companies do not appear within the top the patent applicants, however, when we look to the bigger picture, four of the major players in the pharmaceutical industry are found within the top 50: Janssen Pharmaceutical (no. 20, 29 documents), Bristol Myers Squibb (no. 22, 27 documents), Roche (no. 19, 29 documents), and Pfizer (no. 50, 15 documents). Bayer, GlaxoSmithKline, Sanofi, Abbott Laboratories, and AstraZeneca also are listed with less than 10 documents each. All of the above-mentioned companies appear as patent owners or withing the licensing records for the period.

**Figure 4.**
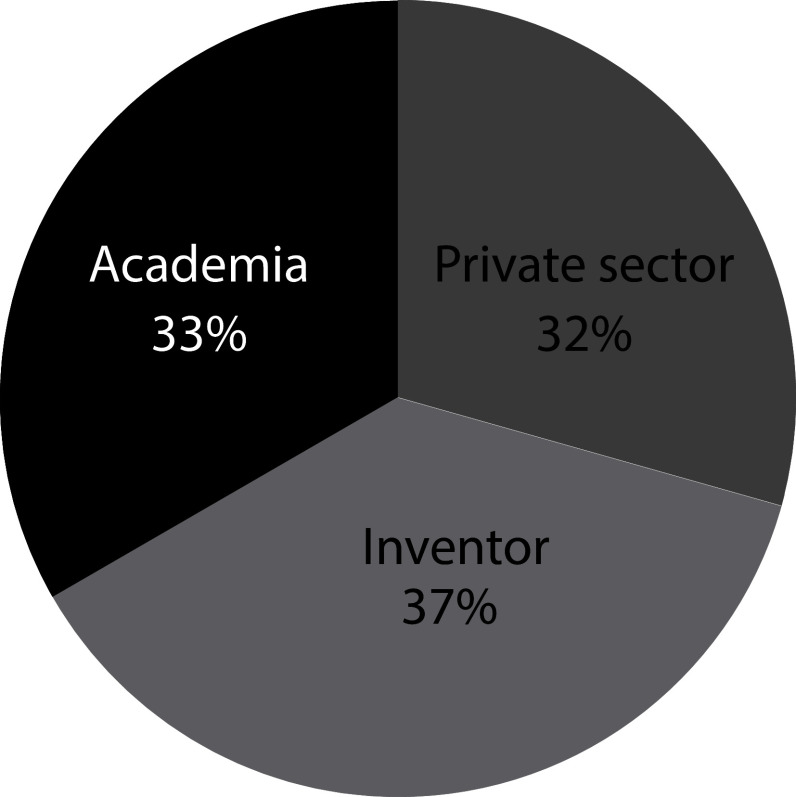
Distribution applicants’ sector of patent documents holders in the field of stem cell therapies during the last decade. Own elaboration with data of The Lens Patents. ^21^

Figure 4 illustrates how patent documents are distributed among holders. 33% of the patent documents are held by Academia-related institutions. The dominant presence of universities in the field suggests that, as stated above, long incubation periods within academia are required for this technology to be ready. These results represent a shift in trends from 2000’s when 56% of the patent ownership has concentrated in private institutions in which only 8% was represented by inventors, and the remaining 44% was held by public governmental institutions and academia. ^19^

**Figure 5.**
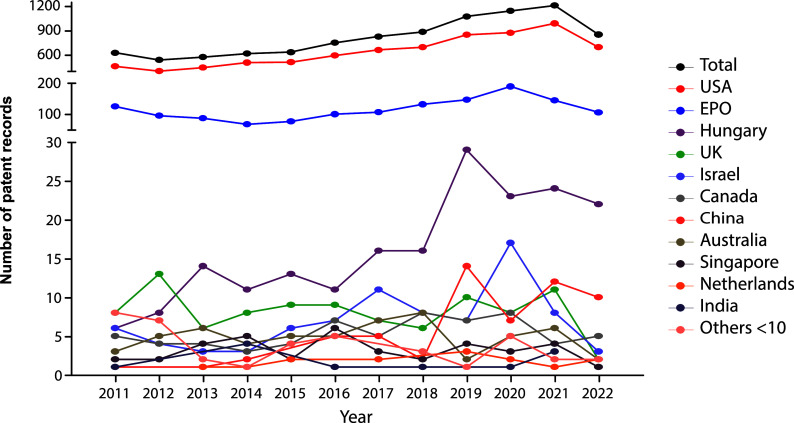
Annual distribution of patent applicants’ sector in the field of stem cell therapies during the last decade. Own elaboration with data of The Lens Patents. ^21^

From 2011 to 2015 the majority of applicants were individuals, however, since 2015 a shift trend is reported when academic institutions and private companies lead the field of patenting activities ( Figure 5). Therefore, technology transfer offices and licensing agreements could be fundamental in bringing stem cell technology to society.

A recent patent analytics report of the Centre for Stem Cell Systems of the University of Melbourne investigated technology development related to mammalian pluripotent stem cells. ^28^ Patent families in this technology are predominately directed at differentiation (28%) and stem cell production (26 %). The high number of patent families granted or with protection being sought (98%) for stimulation and tissue engineering indicates the importance of patent protection in this technology. This analysis showed that innovation in mammalian pluripotent stem cells is dominated by universities and research institutes and has a healthy level of collaboration, which indicates that the technology is in the early stages of development. Nevertheless, it is a growing field with enormous opportunities for translating research into applications as technology matures.

**Figure 6.**
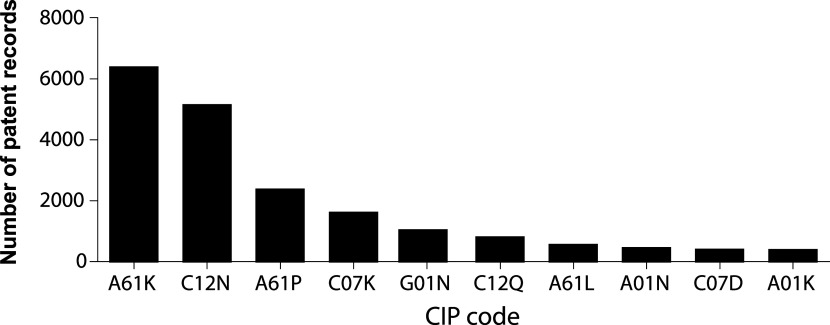
The 10 most frequent IPC codes for patent documents in the field of stem cell therapies during the last decade. Own elaboration with data of The Lens Patents. ^21^

A clear strategy to achieve this migration from bench to bedside can be depicted from analyzing the IPC codes under which inventions are indexed. Figure 6 illustrates the 10 more frequent codes for stem cell patents in the last decade. IPC A61K is assigned to inventions classified as compositions for dentistry, cosmetic, or medical purposes. IPC code C12N encompasses microorganisms, enzymes, and compositions that include substances produced by or derived from, microorganisms or animal cells. A61P IPC classification covers the therapeutic activity of chemical compounds or medicinal preparations already classified as such in subclasses A61K or C12N. Altogether, this classification strategy suggests that potentially commercialized products are assembled medicinal preparations of stem cells, or its derivates.

## How to dilute access to stem cell technologies to achieve social welfare?

Patent systems' primary purpose is to encourage innovation to achieve social welfare by granting exclusive rights over an invention if this fulfills three criteria: novelty, utility, and non-obviousness. Hence, the nature of a patent is to generate profits and gain control over the market, risking its original justification: social benefit. ^10^

Our research shows that innovation in stem cell technology is concentrated within a few hands of the wealthiest sectors: (1) the eight nations with more patent filing activity are listed as high-level income countries by The World Bank, ^8^ (2) nine of the most active organizations in the patenting filed are located at the USA, and (3) four of this top ten are private companies, whose logical-financial-interest for protecting its inventions is to make to most of it. So, how to balance access to stem cell technologies? As the global innovation landscape changes rapidly, a noticeable reshaping of the consumption and use of patent information happens. Brand new technologies, changing business needs, and evolving talent markets continuously affect the patent data's nature, shape, and transformative value. Here we discuss some keystones to consider for bridging the gap.

### Commercialization of innovations is essential to make them affordable

Stem cell and genomic research share controversies around IPR and policy contexts. However, they also have in common the complex and lengthy translational pathways of a complex innovation ecosystem based on working with human materials. ^11^ After that, an essential lesson from genomics can be learned: the progress and use of technology improve its speed and quality while reducing associated costs.

In 2002, after an international 13-year effort, the Human Genome Project satisfactorily concluded, making publicly available 99% of 3000 million bases comprising the whole sequence of human DNA. ^35^ After this breakthrough event, genome sequencing costs decreased by 10,000 times, from 1000 to 0.1 US dollars per megabase from 2007 to 2015. ^36^

Like genomics before the Human Genome Project, stem cell research for therapeutical applications may be unattainable, so reducing the price of this technology becomes essential to impact the health and life of people around the world. A proposed strategy to achieve affordability for stem cell therapies are the stem cells biobanks.

Stem cell biobanks, public or privately funded, ^37^ are highly specialized facilities aiming to collect and store human tissue samples to achieve isolation, storage, and long-term viability of human cells ex vivo. ^38^^,^^39^ Biobanking can be beneficial to reduce costs, personalize therapies, and minimize patient inconvenience to access stem cell lines for both autologous and allogenic settings. ^40^ Ideally, stem cell biobanks will be centralized entities with national and international legal counseling to work under harmonizing ethical protocols and unified international legislation to guaranty the purity and safety of the biorepository to assure the highest quality regarding the stem cell lines offered to interested researchers and clinicians. ^37^^,^^39^^,^^41^

Since early 2010’s, USA, Japan, and European governments (Germany and UK) have been the primarily sponsors for biobanks. ^37^ In USA, majority of biobanks are developed within academic and medical research institutions or tied to public health delivery systems, yet private funded smaller scale and disease-specific biobanks have also proliferated. ^42^

Stem cells biobanks are designed to be scientific-driven entities that provide reliable and high-quality human stem cells to push forward innovative research and novel cell-based therapies by making cellular products readily available. ^38^^,^^39^^,^^43^ However, early biobanking initiatives have been hinder by lack of governance frameworks, the influence of local experience for public trust, and concerns about financial sustainability. ^37^

As he novel regulatory models for cell-based therapies are promising to revive interest in biobanking, probably funded by private institutions, these challenges need to be tackled by drawing clear paths around IPR and developing congruent business model. ^13^^,^^44^ As proposed by Chalmer and colleges: “redefining commercialization activities and focusing on the creation of knowledge rather than commodities realigns commercialization with notions of public good”. ^37^

### Assembly of pre-existing technologies may bring closer science to clinics

A decade ago, the field of Regenerative Medicine – stem cell therapies included - was dominated by small biotechnology companies focused only on tools and nontherapeutic products or their services and manufacturing. ^11^ However, our findings suggest a shift in this behavior, not only because the two medical facilities focused on clinical research rank among the leaders in the field, but, more patents have been filed under more than one category, and selected IPC codes relate to medical preparations and therapeutical activities of compounds or preparations, implying that on-the-edge technologies tend to incorporate different innovations to achieve a therapeutical alternative.

Advanced therapies involve a variety of inventions in many technical fields to manufacture a product, such as tissue selection, cell isolation, purification, culture, and specific therapeutical modifications – lineage differentiation, genetic changes, co-culture, and scaffold assembling-, GMP and quality assurance, transportations strategies, and strategies to transplant into the patient. These are just some of the scientific breakthroughs needed to be assembled through diverse technological developments – potentially patentable - to manufacture a stem cell therapy product. Thus, patent strategies used to protect small-molecule compounds are not likely to work well in the stem cell field; a technology portfolio is essential to cover and protect a commercial product. ^45^

A possible strategy to integrate these patent portfolios and seize opportunities to expand new business areas is to take advantage of the existing technological strengths of different institutions. However, under the current patent systems, these results are unthinkable.

### Alternative strategies for technology transfer

Technology transfer is the span to close the gaps between academic research, industrial applications that allow commercialization of the result, and research's ultimate purpose: social welfare.

Typical licensing agreements had proved wrong for biotechnologies - as stem cells - to be transferred. The enormous fees involved hinder innovation instead of promoting novel developments – as WARF patent licensing fees slowed the advance of stem cells for a timespan. ^46^ Hence, new ventures for technology transfer promise to close the gaps: academic spin-offs to transfer research to industry, corporate spinouts to share technology between private companies, and internal company start-ups to overcome innovation barriers within corporations. ^47^ Nevertheless, endless jigsaws can be arranged based on those approaches.

In the early 2000s, financing stem cell research with strategies based on classic pharmaceutical models resulted in a “disappointing commercial history of cell therapy and contributed to the cool reception stem cell companies receive from venture capitalists”. ^13^ However, the scenery changed after these new models matured: “some of the largest rounds of venture capital ever seen went into 2019 biotech startups”. ^48^

**Figure 7.**
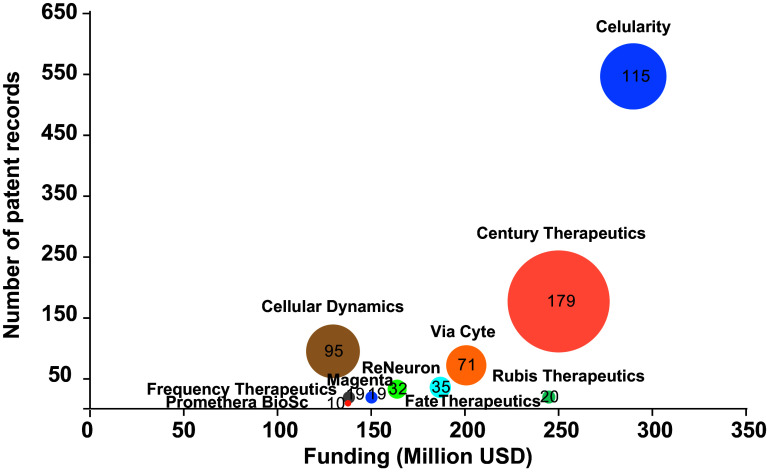
The top ten of stem cell startups. Funding and size of the patent portfolio. The diameter of the bubble represents the number of simple patent families. Own elaboration with data from Lens ^21^ and Medical Startups. ^49^

New stem cell arose start-up companies, whose individual funding accounts for no less than 2 million US dollars, are scattered around the world, with 18 in North America (16 in the USA and two in Canada), seven in the European Union (three in the UK, one in Belgium, Netherlands, Germany, and Switzerland), three at Israel and only one in India. ^49^ By analyzing the behavior in patenting activity of these stem cell-based start-ups, we can give a closer picture of the landscape in this emerging field. The scatter plot in Figure 7 presents patent portfolio size compared to funding amount for the top ten start-ups; funding data was retrieved from Medical Startups ^49^ at the same time, patent information was collected from the Lens patent database ^21^ by searching the number of patent records and families for each patent stakeholder listed (start-ups and associates). The trend shows more funding for start-ups with more solid patent portfolios – more patents with broader cover-, such as Celularity, Century Therapeutics, and Via Cyte; opposite to Rubius Therapeutics, a startup with limited patent families receiving more funding, and Cellular Dynamics, a less funded company with more patent families. Other start-ups listed within the top ten show emerging patent portfolios with more recent patent applications and families, ranging from 1 to 35 patent documents. However, optimistic as this may sound, future challenges will arise when these recently created enterprises consolidate as freedom-to-operate entities with existing patent grants.

Celularity, a $290 million funded Celgene Corporation spinout with a mature patent portfolio, aims to use placenta-derived stem cells as an alternative approach against blood cancer by developing therapies across autoimmune, degenerative disease, immuno-oncology, and functional regeneration. Noteworthy, Celgene Corporation performed as the seventh most productive institution in the stem cell patent filling landscape. ^49^

## Conclusion

Undoubtedly, patents have been a stairway for stem cell research and development to access commercialization; however, the stairs for these products to evolve into therapies that enable social wellbeing are missing in this blueprint.

In certain jurisdictions, due to the lack of congruent regulatory frameworks to use IPR as a protection resource of knowledge, nondisclosure mechanisms of confidentiality and trade secrets are the only, but not preferred, options available to protect innovations in the stem cell field ^9^; relegating patents as outdated mechanisms to bring stem cell technologies into the market. Thence, from this ground, we hypothesize: what if alternative figures of intellectual property for stem cell technology can overcome the current challenges?

Novel business models use stem cells as the cornerstone for cutting-edge technologies – 3D printing, bioprinting, organs on a chip, and genomic edition – that need a whole IPR-protected toolset to tackle real problems of rapid aging societies. As presented before, advanced therapies require a variety of inventions in many technical fields to manufacture a product, and patent portfolios are essential to cover and protect a commercialized product. Unfortunately, these systems may hamper innovation and development of stem cell technologies; thus, to overcome these limitations, the surge of protection strategies through trademarks, utility models, copyright, or creative commons are alternatives that close the gap.

Translating research outputs to economic and social benefits is highly challenging and requires a combination of expertise and bridge builders to connect the research and business worlds. Moreover, the challenge of commercializing or translating research into meaningful therapeutic applications has become even more critical as the global community needs to build momentum toward post-pandemic recovery. Therefore, those with expertise in the field must be proactive and work cohesively to improve their knowledge base. These challenges include constructing a more robust conceptual framework and improved metrics around knowledge transfer. A combination of qualitative research, vehicles that can bring that research to the market, startups, spin-offs, spinouts, SMEs, large enterprises, or other entities, and bridge-builders able to connect the worlds of research and business. ^50^

By analyzing patent activity over the last decade (2011-2020), it becomes evident that limitations notwithstanding stem cells are an area of continuous growth and innovation, evolving in the assembly of technological portfolios to design therapeutic applications patented under diverse IPC codes. The USA, European Union, and Australia are attractive regions for inventive activity protection in the field considering the maturity of their patent systems, leading to patent concentration within a few critical stakeholders with broad coverage through the PCT pathway and concerns about governance regulation, future risks, and inequality. Critical elements are required to build bridges from research to market in a post-pandemic recovery where the global community needs to create momentum. Regulatory updates, novel financing models, new vehicles (start-ups, spinouts, and spin-offs), and alternative figures of intellectual property (nondisclosure mechanisms, trademarks, utility models, copyright, or creative commons) shape plausible avenues in the field of IPR achieve stem cell therapies' ultimate goal, boost wealthiness and welfare in society.

## Data availability

### Source data

Patent data was obtained from WIPO’s patent database Patentscope ( https://patentscope.wipo.int/search/es/search.jsf) on March 2021 through the Advanced Search tool with the query: “CL: ((stem cell* NEAR10 (treat* OR transplant*)) ANDNOT ALL: (“plant” OR “vegetal”))”.
