# Supplementary material for: The patent landscape in the field of stem cell therapy: closing the gap between research and clinic
Source: F1000Res. 2023 Dec 7;11:997. Originally published 2022 Sep 6. [Version 4] doi: 10.12688/f1000research.123799.4 (PMC10933573; doi:10.12688/f1000research.123799.4)
Supplement: Peer review history

# Reviewer report: Reviewer response for version 4

**DOI:** 10.5256/f1000research.159307.r228513

**Published:** 2024-03-12

**Authors:** Pratap Devarapalli

**Affiliations:** 1 The University of Queensland, Saint Lucia, Queensland, Australia

The updated version of the article reflects the suggested changes and no further changes are required.

Is the topic of the opinion article discussed accurately in the context of the current literature?

Partly

Are arguments sufficiently supported by evidence from the published literature?

Yes

Are all factual statements correct and adequately supported by citations?

Yes

Are the conclusions drawn balanced and justified on the basis of the presented arguments?

Partly

Reviewer Expertise:

Patent law, Patent analytics, Genomics, Synthetic biology

I confirm that I have read this submission and believe that I have an appropriate level of expertise to confirm that it is of an acceptable scientific standard.

# Reviewer report: Reviewer response for version 3

**DOI:** 10.5256/f1000research.154016.r197985

**Published:** 2023-08-29

**Authors:** Pratap Devarapalli

**Affiliations:** 1 The University of Queensland, Saint Lucia, Queensland, Australia

I have carefully read the new version and compared it with the previous one. I have also checked the authors’ detailed responses to my previous report.

 I am pleased to see that the authors have addressed most of my suggestions and improved the quality and clarity of their article. They have provided more specific examples and case studies, discussed some landmark cases, provided more details on key players and areas of innovation, and rewritten some sentences to correct the language.

 However, there are still some issues that I think need further attention. The authors have not provided any evidence or reasoning to support their claims about the role and impact of patenting in stem cell therapy. They may also need to provide a nuanced discussion on the potential risks and drawbacks of each strategy, such as legal disputes, ethical dilemmas, market failures or social inequalities. The suggested citation is very relevant to the current article, however, the authors have also not considered citing the suggested article in the revised version.

 Therefore, I would like to maintain my previous status of Approved with reservations for this version of the article. I believe the article could be further improved by addressing these remaining issues.

Is the topic of the opinion article discussed accurately in the context of the current literature?

Partly

Are arguments sufficiently supported by evidence from the published literature?

Yes

Are all factual statements correct and adequately supported by citations?

Yes

Are the conclusions drawn balanced and justified on the basis of the presented arguments?

Partly

Reviewer Expertise:

NA

I confirm that I have read this submission and believe that I have an appropriate level of expertise to confirm that it is of an acceptable scientific standard, however I have significant reservations, as outlined above.

# Response

**Published:** 2023-11-27

**Authors:** Dinorah Hernandez-Melchor

**Affiliations:** Centro de Investigación y de Estudios Avanzados del Instituto Politécnico Nacional, Mexico

Dr. Pratap Devarapalli

*Reviewer for F1000Research Journal*

 We have carefully considered the points raised by the referee to strengthen our work. A point-by-point insight into our answers to the reviewer's suggestions is detailed:

**Point 1. Provide further reasoning and evidence around the role and impact of patenting in stem cell therapy and a nuanced discussion on the potential risks and drawbacks of alternative IPR.**

 As suggested by the reviewer, we further discussed the potential impact of patenting activity by providing, within the section “Alternative strategies for technology transfer,” reasoning on patenting benefits and drawbacks based on the data presented in the work—also, risks and drawbacks of alternative forms of IPR. Furthermore, within the scope of our work for finding new approaches to technology transfer to overcome the hindering of innovation within stem cell therapies due to external factors, we included a discussion around a model that may allow the evaluation of those risks.

 Further evidence on this phenomenon has yet to be established as these new models will be influenced by national policies driving its market reach and the acceptance of those as therapeutic strategies. The evolution of how potential risks and drawbacks impact the stem cell therapy field is within the scope of our future research.

**Point 2. Suggested citation by the reviewer.**

 In the suggested citation, Pratap Devarapalli (the reviewer) and colleagues provide inputs on technological patent trends around iPSC technology a decade after their creation, describing three relevant areas: 1) diseases and treatments that could potentially benefit from the inclusion of iPSC technology, 2) portfolio size of some of the top assignees for iPSC-related patents in the field eight years ago (by 2015), and 3) Patent-Product Linkage to stylish the patent portfolio size required for the development of a product that has reached the market.

 We included the suggested citation within the section “Stem cell therapeutic applications are an area of continuous growth and innovation” as a reference for the reader towards a deeper understanding of technological patent trends around iPSC technology a decade after their creation (2006-2015).

 We hope the referee finds our response adequate and that the revised version is now suitable for indexing.

 Best regards,

 the authors

# Reviewer report: Reviewer response for version 2

**DOI:** 10.5256/f1000research.141283.r177296

**Published:** 2023-06-12

**Authors:** Pratap Devarapalli

**Affiliations:** 1 The University of Queensland, Saint Lucia, Queensland, Australia

The article provides a comprehensive overview of the patent landscape in the field of stem cell therapy. It discusses the implications of this landscape for closing the gap between research and clinical practice. The authors argue that patenting stem cell technologies is essential for fostering innovation and ensuring access to safe and effective therapies. However, they also acknowledge the challenges and controversies that arise from ethical, legal, and social issues.

**Suggestions:**

 The article is well-written and informative. However, there are a few minor suggestions that could be made to improve it.

- **Provide more specific examples and case studies.** The authors could provide more specific examples and case studies to illustrate their points and support their arguments. For example, in section 1, they could mention some examples of clinical trials or approved therapies using different types of stem cells, such as hematopoietic stem cell transplantation for blood disorders, mesenchymal stem cell therapy for inflammatory diseases, or retinal pigment epithelium derived from iPSCs for age-related macular degeneration.
- **Weaving a discussion around landmark cases relevant to stem cells.** The authors could discuss some landmark cases that have shaped the patent landscape in each region. For example, they could discuss the *Brüstle v Greenpeace* case in Europe that ruled out the patentability of human embryonic stem cells, the *WARF v Consumer Watchdog* case in the United States that upheld the patentability of human embryonic stem cells, the CRISPR-Cas9 patent dispute between *UC Berkeley and Broad Institute *that involved gene editing of iPSCs, or the *iPS Academia Japan v Cellular Dynamics International* case in Japan that involved licensing agreements for iPSC technologies.
- **Provide more details on key players and areas of innovation.** The authors could provide more details on some key players or areas of innovation in stem cell patenting. For example, they could discuss the role and impact of public institutions, such as universities, research institutes and government agencies, in generating and disseminating stem cell knowledge and technologies. They could also discuss the emerging fields or applications of stem cell therapy, such as organoids, bioengineering, gene therapy and immunotherapy.
- **Provide evidence or reasoning to support claims.** The authors could provide some evidence or reasoning to support their claims about the role and impact of patenting in stem cell therapy. They could also consider some potential risks and drawbacks of each strategy, such as legal disputes, ethical dilemmas, market failures or social inequalities.

**Suggested Citation:**

 Devarapalli, P., Bhalke, S.L., Dharmadhikari, N.S., Mishra, V., Mago, N., Deshpande, N. and Hirwani, R., 2016. Induced pluripotent stem cells: inventors turning into competitors. *Journal of Commercial Biotechnology*, *22*(4).

**Language:**

 The article is generally well-written and clear, but there are some minor grammatical or language mistakes that could be corrected. Here are some examples:

- In the abstract, the sentence “Patent eligibility of stem cells has been controversial as restrictions to access the fundamental technologies open a gap between research and clinic.” should be rewritten as “Patent eligibility of stem cells has been controversial as restrictions to access the fundamental technologies have opened a gap between research and clinic.”
- In section 1, the sentence “The main advantage of these cells is that they can be obtained from different sources such as bone marrow, adipose tissue, umbilical cord blood and dental pulp.” should be rewritten as “The main advantage of these cells is that they can be obtained from different sources such as bone marrow, adipose tissue, umbilical cord blood or dental pulp.”
- In section 2, the sentence “In this context, patenting is essential for fostering innovation and ensuring access to safe and effective therapies.” should be rewritten as “In this context, patenting is considered essential for fostering innovation and ensuring access to safe and effective therapies.”
- In section 3, the sentence “The United States is the country with more patent applications in this field (Figure 2), followed by China and Japan.” should be rewritten as “The United States is the country with the most patent applications in this field (Figure 2), followed by China and Japan.”
- In section 4, the sentence “This approach would allow sharing knowledge and resources among different actors in the field.” should be rewritten as “This approach would allow the sharing of knowledge and resources among different actors in the field.”

Overall, the article provides a comprehensive overview of the patent landscape in stem cell therapy. It discusses the implications of this landscape for closing the gap between research and clinical practice. The article is well-written, informative, and timely. It demonstrates the authors' expertise and knowledge on the topic. It is also engaging and accessible to a wide audience. The article provides some useful recommendations and perspectives on how to foster a more harmonized, transparent, and collaborative patent system that can facilitate knowledge transfer, technology development, and clinical translation.

Is the topic of the opinion article discussed accurately in the context of the current literature?

Partly

Are arguments sufficiently supported by evidence from the published literature?

Yes

Are all factual statements correct and adequately supported by citations?

Yes

Are the conclusions drawn balanced and justified on the basis of the presented arguments?

Partly

Reviewer Expertise:

Patent law, Patent analytics, Genomics, Synthetic biology

I confirm that I have read this submission and believe that I have an appropriate level of expertise to confirm that it is of an acceptable scientific standard, however I have significant reservations, as outlined above.

## References

[1] : Induced Pluripotent Stem Cells: Inventors turning into competitors. *Journal of Commercial Biotechnology* .2017;22(4) : 10.5912/jcb756 10.5912/jcb756

# Response

**Published:** 2023-07-31

**Authors:** Dinorah Hernandez-Melchor

**Affiliations:** Centro de Investigación y de Estudios Avanzados del Instituto Politécnico Nacional, Mexico

**Dr. Pratap Devarapalli**

**Reviewer for F1000Research Journal**

 We appreciate the reviewer's opinion regarding the relevance and pertinence of our work and thank for the insightful comments to improve the quality of the manuscript. We have carefully considered the valuable points raised by the referee, and a point-by-point insight on our answers to the reviewer's suggestions points are detailed:

***Point 1: Provide more specific examples and case studies.***

 In the introduction, we added examples of relevant therapeutical applications for different stem cell lineages at both clinical trial and market approval stages to emphasize the clinical potential of these platforms and the consequent interest in protecting IPR in the field.

***Point 2: Weaving a discussion around landmark cases relevant to stem cells.***

 To better illustrate how notable cases influence the patent landscape in different regions and periods, we included within the section “In the patent arena, regulation matters” a discussion on how the patentability of the hESC in the US and Europe during the first decade of the 2000s was shaped by two landmark cases: WARF vs. Consumer Watchdog, and Brüstle vs. Greenpeace case, respectively. Also, we argue how CRISPR/Cas9 and iPSC patents are shaping the future of IPRs in the therapeutical stem cell applications in the inevitable sought for new technological platforms to reach the market caused in the section “Stem cell therapeutic applications are an area of continuous growth and innovation.”

***Point 3: Provide more details on key players and areas of innovation.***

 In the section “Universities and private companies are the leading players in the field”, we included a description of the critical players in the patent arena of stem cell technology and a discussion about the roles they are taking on generating and disseminating stem cell knowledge and technologies based on the reported patent documents data.

 We agree with the referee that contextualizing the reader in the emerging fields and applications of stem cells therapy is relevant; however, as a detailed discussion on these next-generation strategies is beyond the scope of this paper, we included in Section “Assembly of pre-existing technologies may bring closer science to clinics” a reference for an up-to-date comprehensive review on this area by Bashor and colleagues (2022).

***Point 4: Provide evidence or reasoning to support claims.***

 We discussed potential risks and drawbacks for the proposed novel models for the technology transference section in the“Alternative strategies for technology transfer” section.

***Point 5: Language***

 As recommended by the reviewer, we revised and corrected grammatical and language mistakes.

 We hope the referee finds our response adequate and that the revised version is now suitable for indexing.

 Kind regards,

# Reviewer report: Reviewer response for version 2

**DOI:** 10.5256/f1000research.141283.r158806

**Published:** 2023-01-16

**Authors:** Verónica A. Palma

**Affiliations:** 1 Laboratory of Stem Cells and Developmental Biology, Department of Biology, Faculty of Sciences, Universidad de Chile, Santiago, Chile

I have read the new version. The revised version fulfills my concerns, the paper has improved significantly. In particular, the new figures and the new discussion section have addressed the criticisms I'd raised. 

 That said, I approve the manuscript.

Is the topic of the opinion article discussed accurately in the context of the current literature?

Yes

Are arguments sufficiently supported by evidence from the published literature?

Partly

Are all factual statements correct and adequately supported by citations?

Yes

Are the conclusions drawn balanced and justified on the basis of the presented arguments?

Yes

Reviewer Expertise:

Stem cells, regenerative medicine, cancer, developmental biology

I confirm that I have read this submission and believe that I have an appropriate level of expertise to confirm that it is of an acceptable scientific standard.

# Reviewer report: Reviewer response for version 1

**DOI:** 10.5256/f1000research.135938.r149831

**Published:** 2022-10-03

**Authors:** Verónica A. Palma

**Affiliations:** 1 Laboratory of Stem Cells and Developmental Biology, Department of Biology, Faculty of Sciences, Universidad de Chile, Santiago, Chile

The review by Hernandez-Melchor *et al.* is an interesting opinion article and includes novel approaches. The authors present the total number of patent documents in the last decade (2011-2020) and aim to elucidate which actors can be drivers in the research and clinical field in eight countries. As stem cell therapies are increasingly heading to the clinic, there is a substantial need for the issues discussed within the manuscript i.e. intellectual property rights and their implications for stem cell technology transfer to society. Hence, the subject of the review is interesting and could be significant and of potential interest to researchers in the area.

 Therefore, the study addresses a highly relevant topic and the reviewer thinks there is merit in this paper. But the review is insufficient in its current presentation, it is not offering an optimal advance in the field and, as such, the review is not suitable for indexing unless edited.

 As a general comment, the reviewer believes that some of the data presented do not adequately convey the ideas discussed. Also, despite including most of the available bibliography on the topic, some important ones are missing (see for instance one suggested reference, such as Takashima *et al*., 2021 ^1^) and the authors fail to make a good discussion considering their main goal which is intended to promote the clinical development of stem cell-based interventions and technological transfer to achieve welfare in society.

 The following are some suggestions that may be of use to the authors to improve the quality of the manuscript.

 Comments:

- Point 1: Figure 1 shows patent documents in stem cell therapies from 2011 to 2020, revealing an increase in the number of patents since 2015 with its peak in 2019. However, to establish a better understanding of the main trends in stem cell patenting activity, it is necessary and favorable to include a detailed approach to the annual stem cell patent applications and grants.
- Point 2: Figure 2 shows the core collection of US, EPO, and PCT documents and eight countries (2011 to 2020). However, to study the evolution of stem cell patent documents, it is necessary to compare this distribution with the data between 2001 to 2010 to check the atomization or concentration of these data over time.
- Point 3: Figure 3 displays the distribution of ownership between the private sector and public sector entities in patents on stem cell therapies. Nevertheless, it would be interesting to compare with data from 2001 to 2010 even though stem cells are still an early-stage technology due to the high percentage involving public sector organizations.
- Point 4: Page 7 paragraphs a relationship with technology transfer and commercialization of the results to achieve welfare in society. The reviewer suggests exemplifying the technology transfer of the stem cell to the community, describing in particular: How are the public biobanks of stem cells funded in developed and developing countries? How does the existence of public banks in these countries improve patients' quality of life? This would be a clear example of what the authors try to convey.
- Point 5: It is recommended to indicate whether the companies listed in table 1 have undergone M&A by larger companies. Surprisingly the larger pharmaceutical companies are not prominent players in stem cell research. Please refer to this point in more detail.
- Point 6: On page 7 paragraph 5, under the newest version of International Patent Classification (IPC), it would be interesting to report the distribution of patent documents under the IPC code.
- Point 7: Another worthy and necessary addition to this issue could come from the elaboration on how the regulatory framework in regenerative medicine affects the total number of patent documents in the eight countries.

In conclusion, the review is not suitable for indexing unless edited. It requires minor revisions to be 'Approved'.

Is the topic of the opinion article discussed accurately in the context of the current literature?

Yes

Are arguments sufficiently supported by evidence from the published literature?

Partly

Are all factual statements correct and adequately supported by citations?

Yes

Are the conclusions drawn balanced and justified on the basis of the presented arguments?

Yes

Reviewer Expertise:

Stem cells, regenerative medicine, cancer, developmental biology

I confirm that I have read this submission and believe that I have an appropriate level of expertise to confirm that it is of an acceptable scientific standard, however I have significant reservations, as outlined above.

## References

[1] : Reflection on the enactment and impact of safety laws for regenerative medicine in Japan. *Stem Cell Reports* .2021;16(6) : 10.1016/j.stemcr.2021.04.017 1425-1434 10.1016/j.stemcr.2021.04.017 34019814 PMC8190593

# Response

**Published:** 2022-12-09

**Authors:** Dinorah Hernandez-Melchor

**Affiliations:** Centro de Investigación y de Estudios Avanzados del Instituto Politécnico Nacional, Mexico

**Dear Dr. Palma**

**Reviewer for F1000Research**

 We are pleased to read that the reviewer considers the subject of this article interesting and of potential interest for researchers. We thank the reviewer for the insightful comments to improve the quality of the manuscript and have considered the valuable points raised by the referee. A point-by-point account of our answers to the points raised by the reviewer follows:

***Point 1:*** *Figure 1 shows patent documents in stem cell therapies from 2011 to 2020, revealing an increase in the number of patents since 2015 with its peak in 2019. However, to establish a better understanding of the main trends in stem cell patenting activity, it is necessary and favorable to include a detailed approach to the annual stem cell patent applications and grants.*

 We included patent applications and grants data in Figure 1 to encourage better understanding of the activity in the field.

***Point 2:*** *Figure 2 shows the core collection of US, EPO, and PCT documents and eight countries (2011 to 2020). However, to study the evolution of stem cell patent documents, it is necessary to compare this distribution with the data between 2001 to 2010 to check the atomization or concentration of these data over time.*

 We referred to the distribution reported for the 2000s decade. Furthermore, we include the annual data for each filing route (Figure 2) and national offices (Figure 3) to establish a better understanding of the trends.

***Point 3:*** *Figure 3 displays the distribution of ownership between the private sector and public sector entities in patents on stem cell therapies. Nevertheless, it would be interesting to compare with data from 2001 to 2010 even though stem cells are still an early-stage technology due to the high percentage involving public sector organizations.*

 As in Point 2, the distribution for the 2000s decade is referred to and the data for annual distribution of patent applicants’ sector is presented to clarify the dynamics’ change in the field.

***Point 4:*** *Page 7 paragraphs a relationship with technology transfer and commercialization of the results to achieve welfare in society. The reviewer suggests exemplifying the technology transfer of the stem cell to the community, describing in particular: How are the public biobanks of stem cells funded in developed and developing countries? How does the existence of public banks in these countries improve patients' quality of life? This would be a clear example of what the authors try to convey.*

 We discussed the model of stem cell biobanks as a model to distribute and commercialize stem cell products to achieve social welfare.

***Point 5:*** *It is recommended to indicate whether the companies listed in table 1 have undergone M&A by larger companies. Surprisingly the larger pharmaceutical companies are not prominent players in stem cell research. Please refer to this point in more detail.*

 As recommended by reviewer, the applicable M&A for the companies listed in Table 1 is mentioned. The fact that larger biopharmaceutical companies are not listed within top 10 applicants is stated and the role of these institutions as patent owners or within is referred to, however, further discussion of this is beyond the scope of this review and may be addressed in future research.

***Point 6:*** *On page 7 paragraph 5, under the newest version of International Patent Classification (IPC), it would be interesting to report the distribution of patent documents under the IPC code.*

 We include the distribution of IPC codes in Figure 6.

***Point 7*** *: Another worthy and necessary addition to this issue could come from the elaboration on how the regulatory framework in regenerative medicine affects the total number of patent documents in the eight countries.*

 A description of the regulatory framework for advanced therapies was included and its relevance is discussed within sections *2.3. Territoriality of patent documents' filling: leading countries in the field* and *3.1. Commercialization of innovations is essential to make them affordable.*

 We hope that you find our answers adequate and that the revised version is now suitable for indexing.

 Best regards.
